# Supplementary material for: Peaceful queen succession in the naked mole rat
Source: Sci Adv. 2026 Apr 15;12(16):eaef4157. doi: 10.1126/sciadv.aef4157 (PMC13082316; doi:10.1126/sciadv.aef4157)
Supplement: Supplementary file 1 — Figs. S1 and S2 Table S1 [file sciadv.aef4157_sm.pdf]

Science Advances
AAAS

Supplementary Materials for

Peaceful queen succession in the naked mole rat

Shanes C. Abeywardena *et al.*

Corresponding author: Janelle S. Ayres, jayres@salk.edu

Sci. Adv. **12**, eaef4157 (2026)
DOI: 10.1126/sciadv.aef4157

This PDF file includes:

Figs. S1 and S2
Table S1

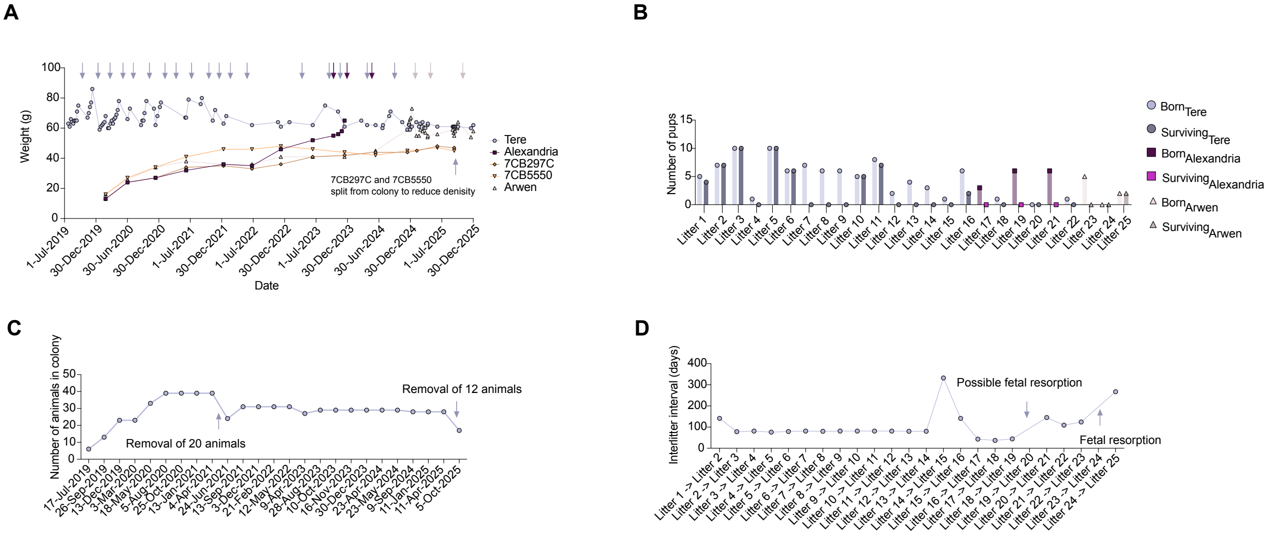

Supplemental Figure 1. Summary of study data. (**A**) Body weight of Queen Teré and four of her daughters born in Queen Teré's December 13, 2019 litter, including Alexandria and Arwen. Blue arrows indicate Queen Teré's litters. Purple arrows indicate Alexandria's litters and pink arrows indicate Arwen's litters. (**B**) The number of pups born and the number of surviving pups past one month of age for the indicated litters. (**C**) The number of animals in the colony one month post noted litter dates. (**D**) Interlitter interval days between the indicated litters for the colony. Data shown in panels were collected from a single captive colony (N=1) over ~6.5 years. Each point represents an individual (**A**) body weight, (**B**) litter, (**C**) interlitter interval or (**D**) colony density. No statistical tests comparing independent biological replicates were performed for (**A-D**) because only one colony was studied. All raw data points are shown. Data from each panel are also displayed in **Figures 2-5**.

Supplemental Figure 2

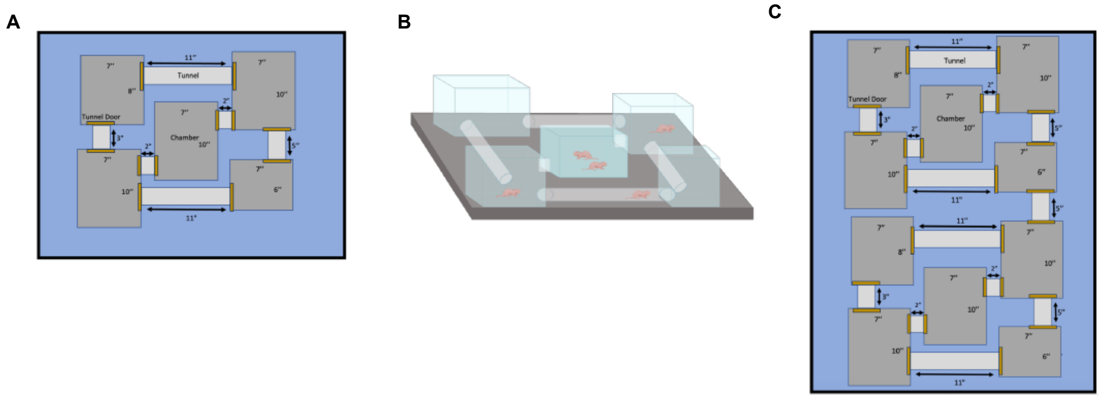

Supplemental Figure 2. Housing used in this study. (A) 5 Chamber housing System. Chambers are in dark gray and range in dimensions of 7”W x 10”L, 7”W x 8”L, 7”W x 6”L. The height of all chambers is 7”. Tunnels are in light gray and range in lengths of 11”, 5”, 3”, and 2.” (**B**) 3D Model of 5 Chamber housing System. Image created in Biorender: AYRES, J. (2026) https://BioRender.com/shc56q5. (**C**) 10 Chamber housing System. Chambers are in dark gray and range in dimensions of 7”W x 10”L, 7”W x 8”L, 7”W x 6”L. The height of all chambers is 7”. Tunnels are in light gray and range in lengths of 11”, 5”, 3”, and 2.”

Animal ID	Birthdate	Sex	Date removed	Reason for Removal
7AA94B0	9/26/19	F	6/21/21	Reduce Amigo colony density
7CB643C	12/13/19	M	6/21/21	Reduce Amigo colony density
7CB659D	12/13/19	M	6/21/21	Reduce Amigo colony density
7CB6D80	12/13/19	F	6/21/21	Reduce Amigo colony density
7CB61CB	12/13/19	M	6/21/21	Reduce Amigo colony density
7CB273C	12/13/19	F	6/21/21	Reduce Amigo colony density
7CB6941	5/18/20	M	6/21/21	Reduce Amigo colony density
7CB6694	5/18/20	M	6/21/21	Reduce Amigo colony density
7CB480F	5/18/20	M	6/21/21	Reduce Amigo colony density
7AAA1E6	5/18/20	F	6/21/21	Reduce Amigo colony density
7AAA8CBB	8/5/20	F	6/21/21	Reduce Amigo colony density
7AA82F0	8/5/20	M	6/21/21	Reduce Amigo colony density
7AAA458	8/5/20	M	6/21/21	Reduce Amigo colony density
7BF324C	8/5/20	F	6/21/21	Reduce Amigo colony density
7CB64B6	12/13/19	F	6/21/21	Reduce Amigo colony density
7CB4467	5/18/20	F	6/21/21	Reduce Amigo colony density
7CB616F	5/18/20	F	6/21/21	Reduce Amigo colony density
7AA8F6A	7/18/19	F	6/21/21	Reduce Amigo colony density
7AAABAC	9/26/19	M	6/21/21	Reduce Amigo colony density
7BDC332	9/26/19	M	6/21/21	Reduce Amigo colony density
Garbanzo	9/13/21	F	7/8/22	Start a new family
7BDC31E	5/18/20	F	7/8/22	Start a new family
Reine	7/18/19	F	7/15/22	Start a new family
7BD93DA	9/13/21	M	8/18/22	Missing - suspected escape
Alexandria	12/13/19	F	9/9/24	Euthanized - uterine torsion
7BDC323	7/18/19	F	6/30/25	Found dead - no fight wounds, nothing remarkable noted in autopsy. Cecum distended, postmortem change?
7AAA327	9/26/19	F	9/11/25	Reduce Amigo colony density
Bruiser	9/26/19	F	9/12/25	Reduce Amigo colony density
7CB297C	12/13/19	F	9/13/25	Reduce Amigo colony density
7CB5550	12/13/19	F	9/14/25	Reduce Amigo colony density
7BF31A7	9/26/19	M	9/15/25	Reduce Amigo colony density
7AAB632	8/5/20	M	9/16/25	Reduce Amigo colony density
Pogo	6/24/21	F	9/17/25	Reduce Amigo colony density
7BDEAA0	6/24/21	F	9/18/25	Reduce Amigo colony density
7BDC047	6/24/21	F	9/19/25	Reduce Amigo colony density
7BDE7C2	6/24/21	M	9/20/25	Reduce Amigo colony density
7BF2BCC	9/13/21	M	9/21/25	Reduce Amigo colony density
7BDECB6	9/13/21	M	9/22/25	Reduce Amigo colony density

Supplemental Table 1. Summary of animals removed from the Amigos colony over the course of the study
